# Azathioprine and aspirin in treatment of childhood primary arterial stroke: therapeutic benefits and side effects

**DOI:** 10.1186/cc10908

**Published:** 2012-03-20

**Authors:** A Alhaboob, G Ahmed

**Affiliations:** 1 King Khalid University Hospital and College of Medicine, King Saud University, Riyadh, Saudi Arabia

## Introduction

The objectives were to describe a cohort of children presenting with medium/large vessel childhood primary angiitis of the central nervous system (PACNS); to report their short-term neurological outcome; and to evaluate efficacy and safety of implemented management.

## Methods

The study included 68 patients, aged less than 16 years. They had their symptoms within 14 days of admission. They received induction therapy with pulses of intravenous steroids and/or intravenous immunoglobulin followed by maintenance therapy with azathioprine and low-dose aspirin. They were also treated with anticoagulants for 4 weeks along with the induction therapy. They were assessed for; their clinical presentation, disease severity (progressive or nonprogressive), hospital course, adverse effects of the used treatment and outcome. Reports of their neuroimaging studies were also collected.

## Results

Studied patients were 42 (62.76%) boys and 26 (38.23%) girls. Their mean age was 8.5 ± 3.5 years. The commonest presenting symptoms were motor deficit (70%), headache (64%) and fever (20%), while the commonest presenting neurological signs were hemiparesis (60%), seizure 55% (focal 35%, generalized 20%), and decreased level of consciousness (30%). Neuroradiological studies of the brain revealed: ischemic strokes in 50 children (73.5%), hemorrhagic strokes in 10 (14.7%) and ischemic-hemorrhagic lesions in eight (11.8%). Conventional angiography (CA) and/or magnetic resonance angiography (MRA) at the time of admission revealed that 51 (75%) patients had nonprogressive and 17 (25%) had evidence of progressive arteriopathy. Out of the studied patients, 56 (81.5%) survived and 12 (18.5%) died. Male sex, deep coma and intracerebral bleeding causing severe raised intracranial pressure were poor prognostic signs. Survivors were discharged on oral aspirin and 15 of them commenced also on azathioprine. On follow-up it was found that out of the 56 survivors, 11 were normal (19.65%), 14 (25%) had minor disabilities, another 11 (19.65%) had moderate disabilities and 20 (35.7%) had severe disabilities.

## Conclusion

The spectrum of cPACNS includes progressive and non-progressive forms. Characteristic features on presentation may predict later progression and outcome; identify a distinct high-risk cPACNS cohort; and guide the selection of patients for immunosuppressive therapy. Further studies are required to substantiate our findings.

